# Supplementary material for: The evolution and convergence of mutation spectra across mammals
Source: Commun Biol. 2025 May 17;8:763. doi: 10.1038/s42003-025-08181-x (PMC12084637; doi:10.1038/s42003-025-08181-x)
Supplement: Supplementary file 2 — Supplementary Information [file 42003_2025_8181_MOESM2_ESM.pdf]

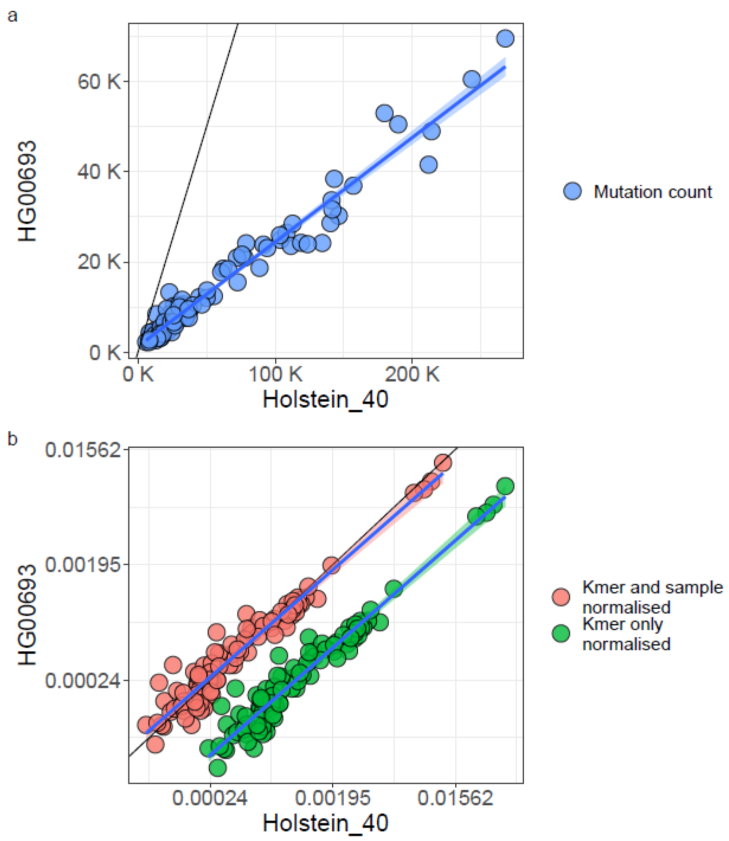

Supplementary Figure 1. Example impact of implemented normalisation approach. A) The raw mutation numbers in a randomly selected human (y axis) and cow (x axis) genome. Each dot represents the frequency of a given 3mer change. The parity line is shown in black. The cow genome carries substantially more mutations than the human genome. B) The impact of normalising by each genome's ancestral 3mer frequency (green) and the impact of further applying the median of ratios approach to each sample (red). Again the parity line is shown in black. Applying both corrections puts the mutation spectra of the two genomes on corresponding scales so that they can be more readily compared.

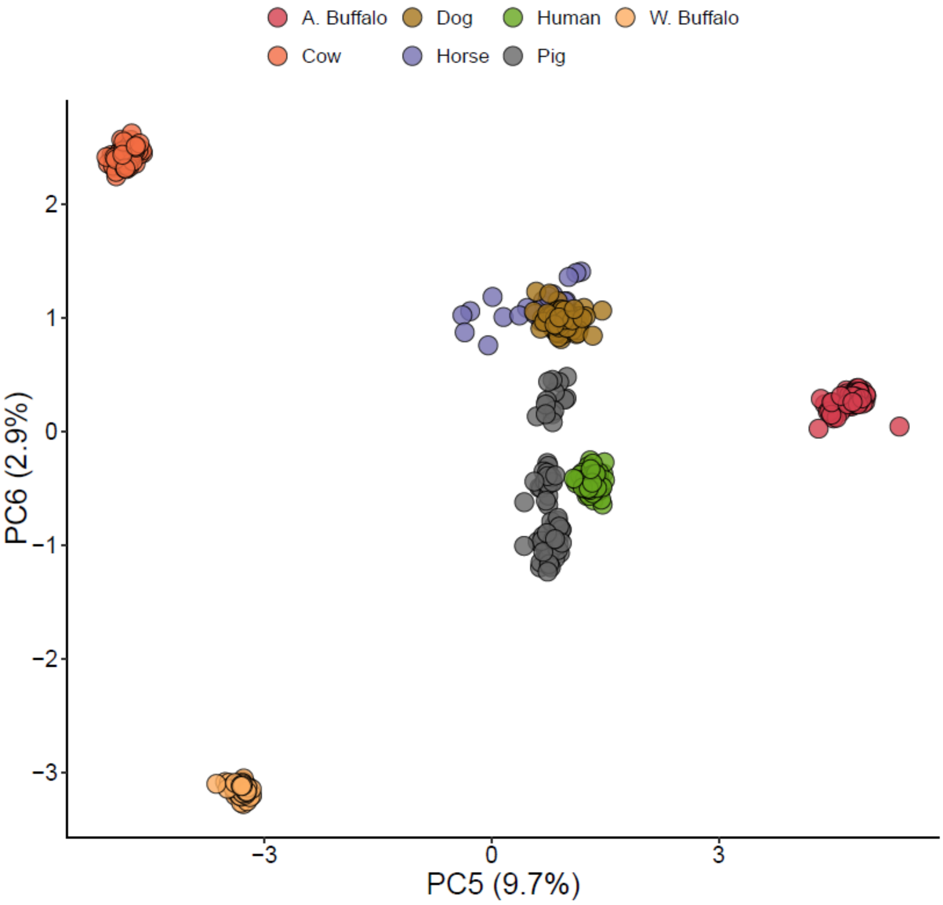

Supplementary Figure 2. Principal component analysis of the relationship between different species based on the rate of SNV mutations of different ancestral 3-mers. PC5 vs PC6 is shown, illustrating how, despite their relatively close evolutionary distance, the Bovidae can be clearly separated by their underlying mutational spectra on these principal components.

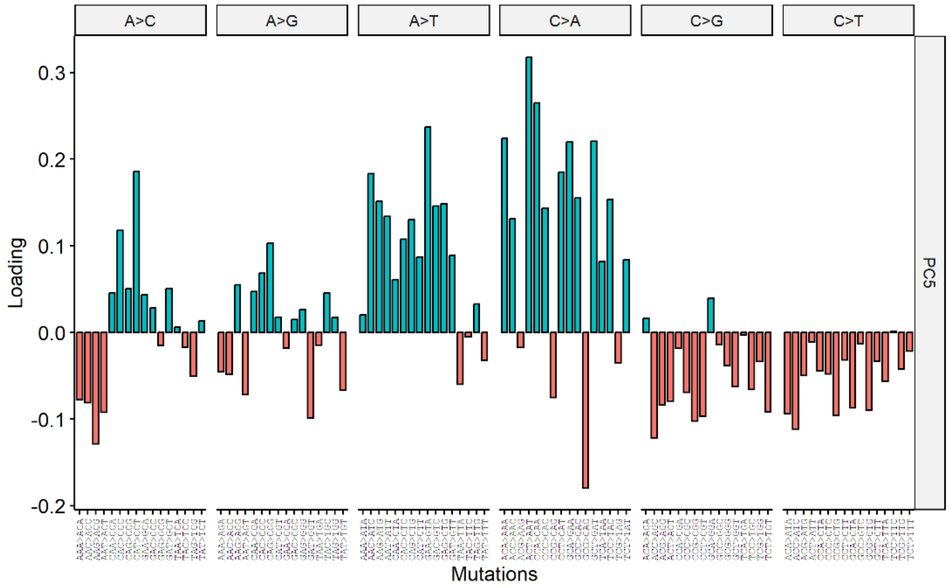

Supplementary Figure 3. Loadings associated with PC5 in the between species SNV mutation profile comparison.

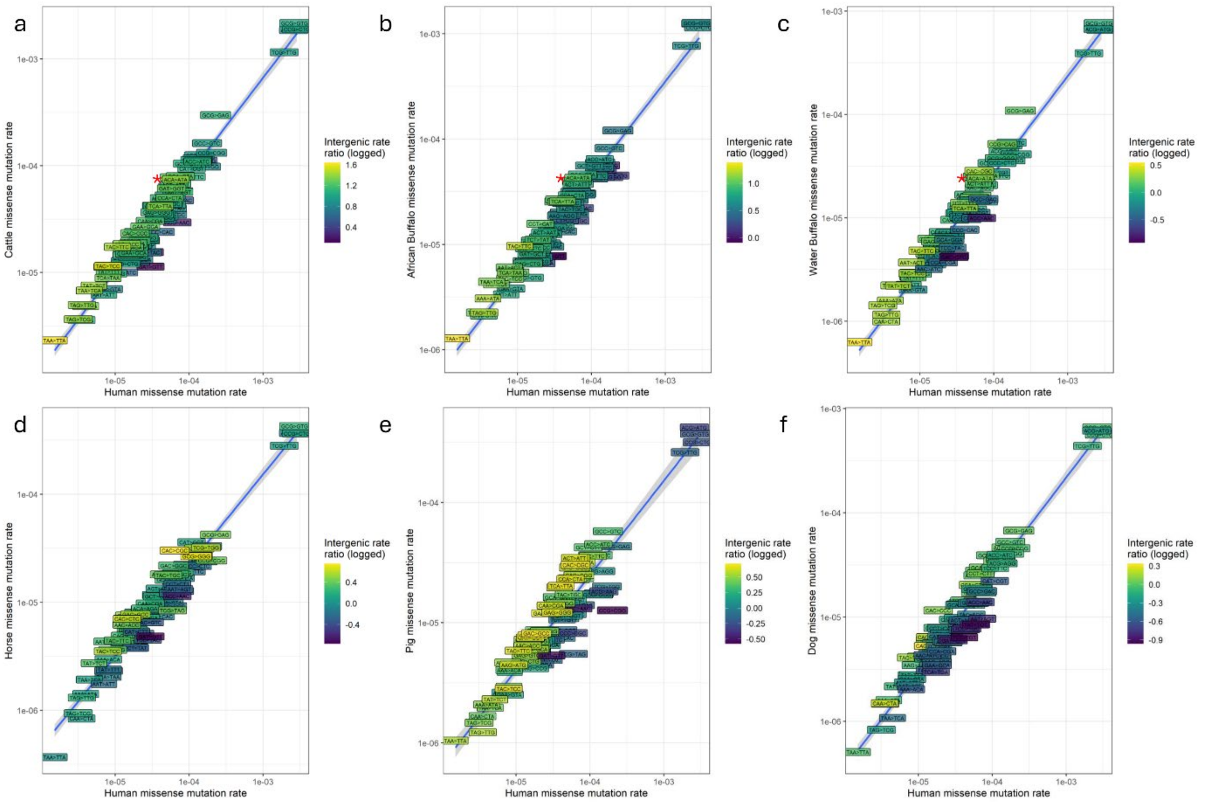

Supplementary Figure 4. The rate of different changes that lead to amino acid changes in humans versus the other size species. The colour of each point corresponds to the ratio of the rate of the same change between the same species, but in intergenic regions. The A[C>T]A change showing elevated rates in Bovids compared to other species is indicated.

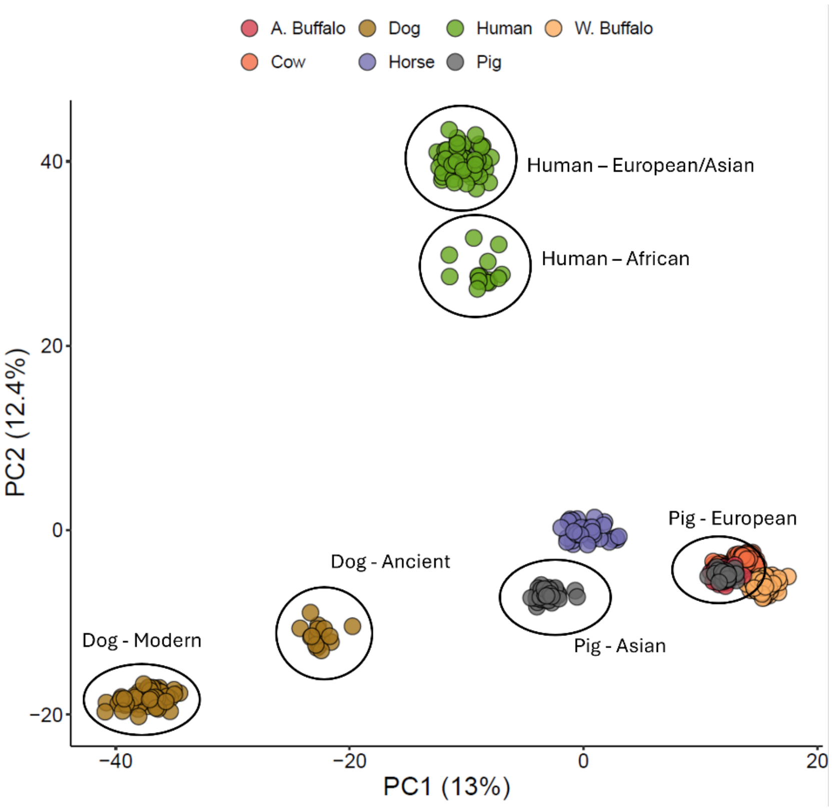

Supplementary Figure 5. Principal component analysis of the relationship between different species based on the rate of SDM mutations of different ancestral 3-mers. The species with larger numbers were randomly downsampled to a maximum of 80 individuals. Further sub-division of the dog, pig and human populations is observed relative to the corresponding PCA for SNVs shown in Figure 2A.

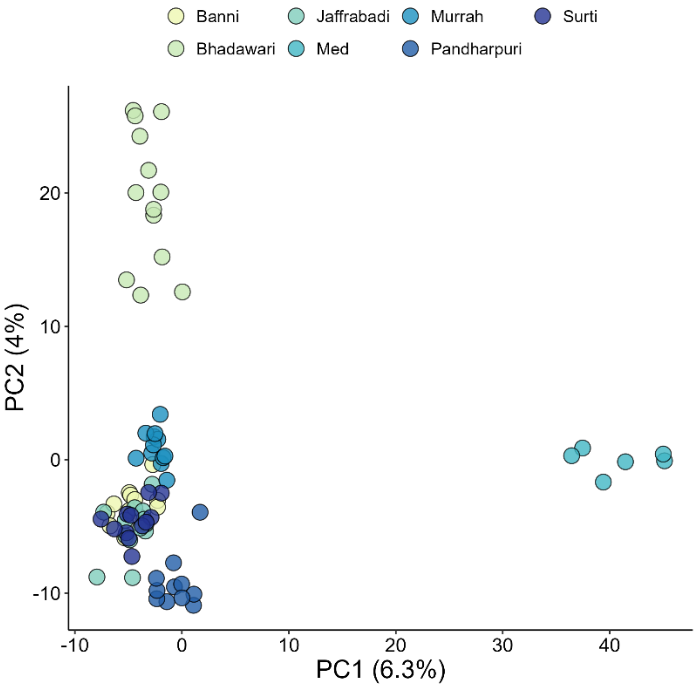

Supplementary Figure 6. Principal component analysis of the relationship between different water buffalo breeds based on the rate of SDM mutations of different ancestral 3-mers.

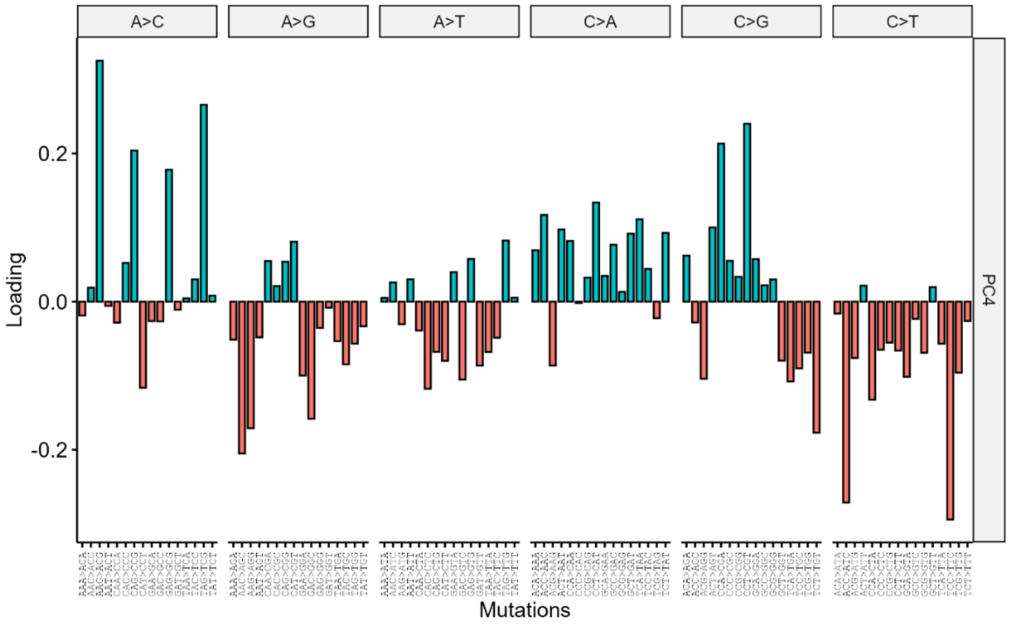

Supplementary Figure 7. Loadings associated with PC4 in the between cattle population SNV mutation profile comparison. Positive loadings indicate a relative enrichment in African indicine populations. Negative loadings a relative enrichment in East Asian indicine.

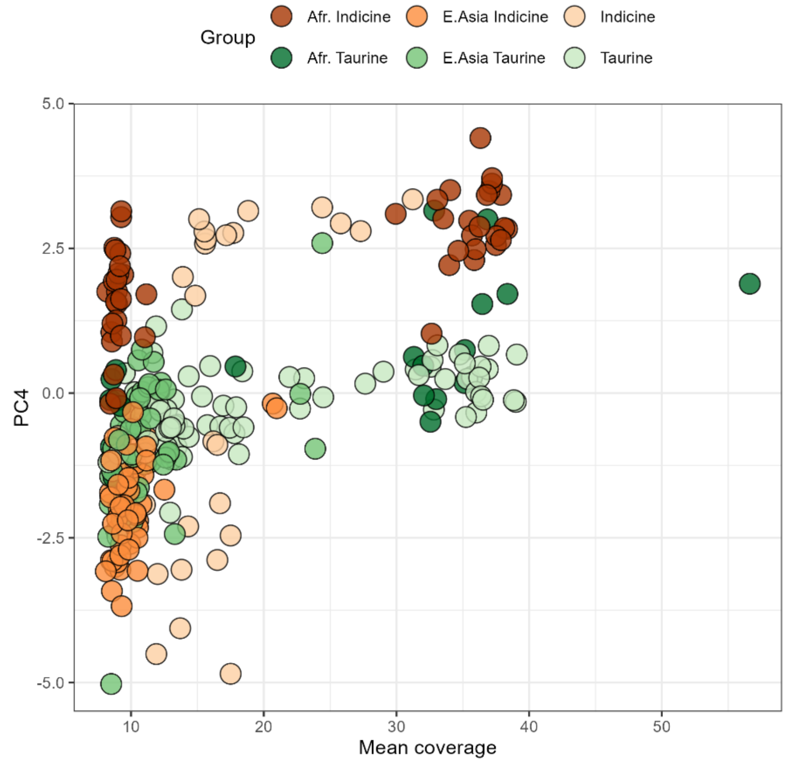

Supplementary Figure 8. Cattle PC4 versus sample coverage. Sample coverage does not appear to be driving the separations observed in PC4. For example, African Indicine cattle have a positive PC4 value irrespective of their depth, with East Asian Indicine cattle showing a negative PC4, despite having similar coverages to many of the African Indicine samples. Likewise the primary Indicine split is observed among samples of similar coverages.

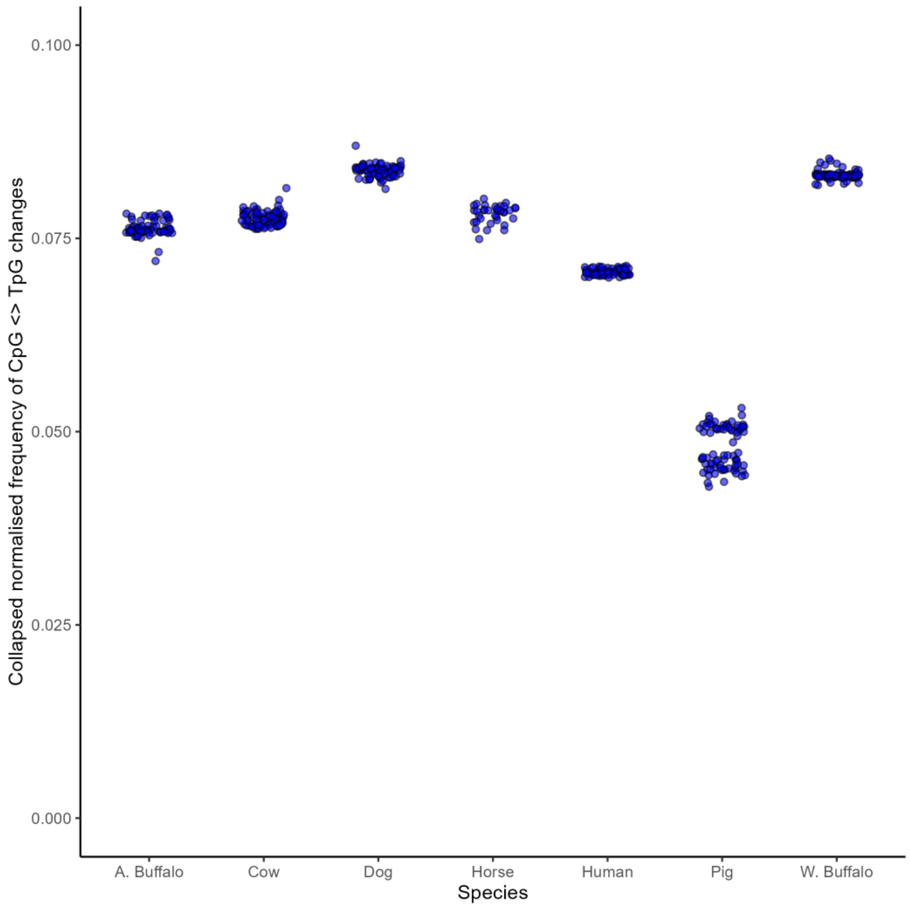

Supplementary Figure 9. The relative frequency of CpG<>TpG changes is lower in pigs, suggesting that the observed depletion of CpG>TpG changes in this species cannot simply be attributed to incorrectly inferring the direction of change at these sites. The split between the Asian and European pigs is also evident.

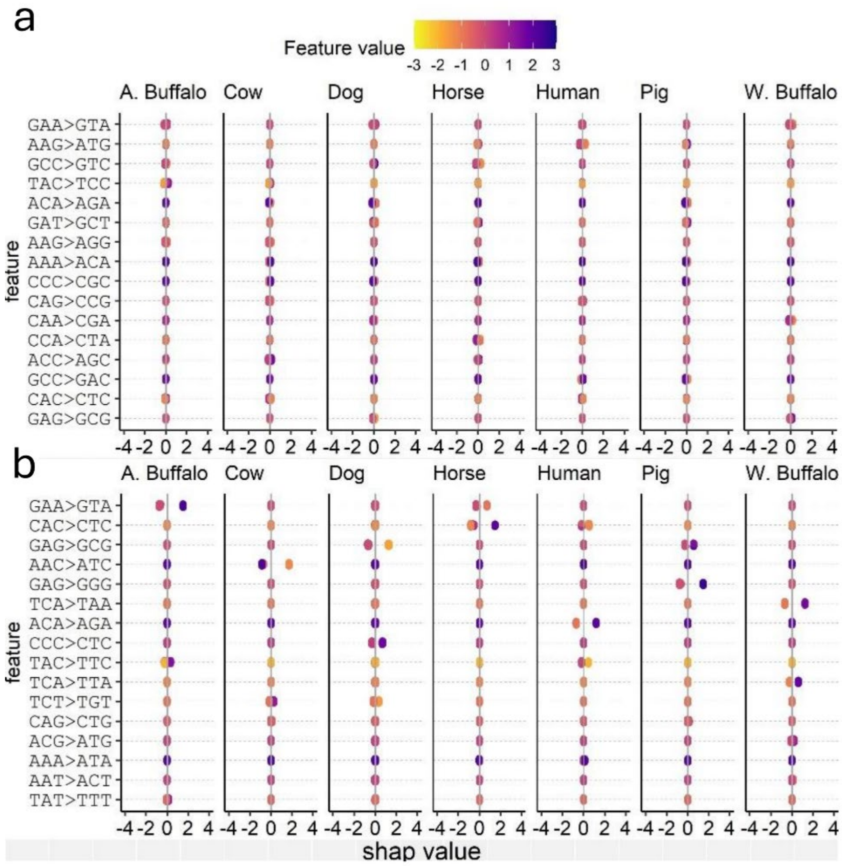

Supplementary Figure 10. SHAP values calculated using a) 10 and b) 30 individuals per species, highlighting the issue of discriminating factors at too small sample sizes.

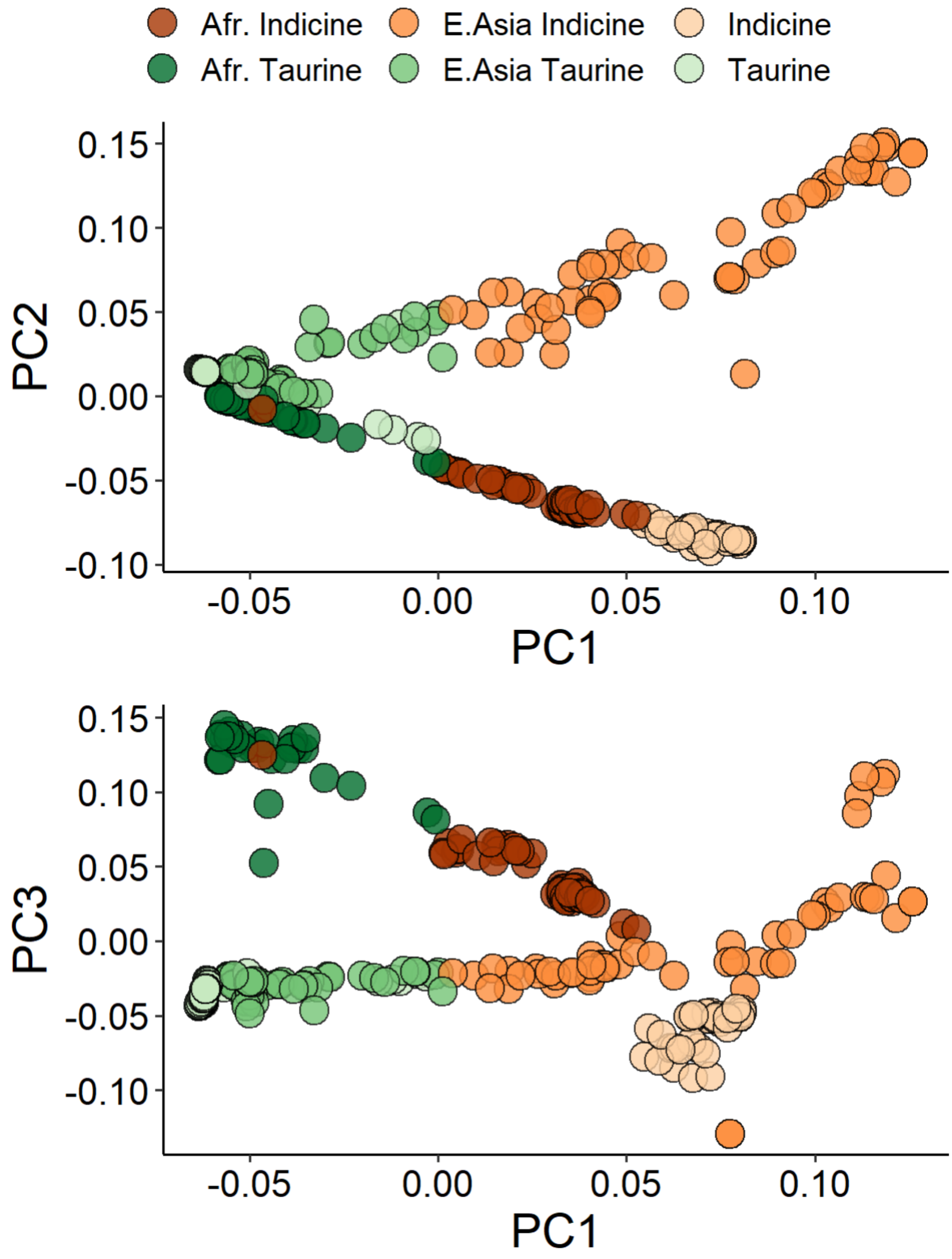

Supplementary Figure 11. Cattle PCA plots based on SNP genotypes (not mutation spectra).

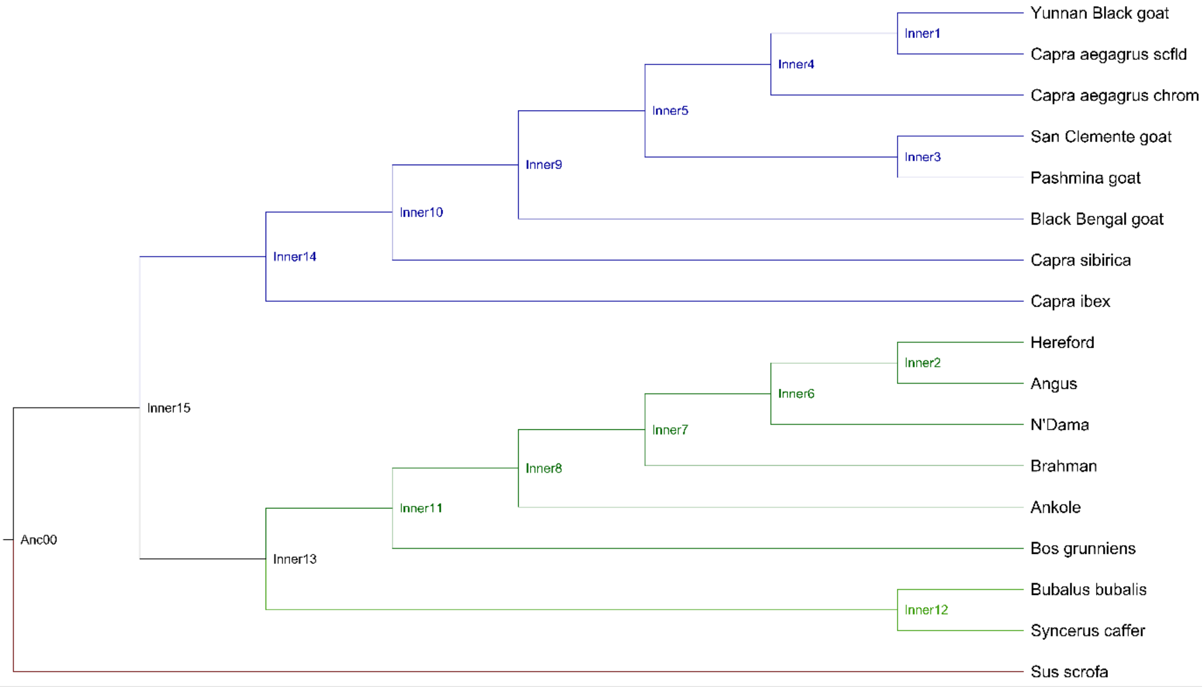

Supplementary Figure 12. The genomes included in the novel progressive cactus alignment in order to derive the bovid ancestral genomes.

Species1	Change	Cattle	Water Buffalo	African Buffalo	Horse	Pig	Dog	Human
Cattle	AAA>ACA	1	0.00121994738728289	0.00927311916742557	NA	NA	5.57570600363696e-09	6.08378858761293e-17
Cattle	AAA>AGA	1	0.000490095606616746	0.117292171054305	NA	5.28573959034731e-08	2.32580472278855e-08	NA
African Buffalo	AAC>ACC	NA	0.141877898132899	1	4.10507284182806e-11	7.83954595909373e-06	1.29269306135979e-06	NA
African Buffalo	AAT>ACT	0.602586197912456	0.546758920306241	1	1.15745038882749e-06	NA	NA	1.3053950642281e-10
Cattle	AAT>ACT	1	0.920382098035559	0.602586197912456	4.16451302599043e-07	NA	NA	1.74788321064987e-13
African Buffalo	AAT>AGT	0.0547136664358482	1.7945933056882e-05	1	NA	3.52473300944351e-06	2.3966774681259e-21	4.02448460482043e-17
Cattle	AAT>AGT	1	NA	0.0547136664358482	NA	5.59886567548005e-06	5.05626152559897e-30	3.31659356992353e-23
Water Buffalo	AAT>AGT	NA	1	1.7945933056882e-05	NA	1.16448830091624e-16	4.19284680552372e-07	1.26306195754008e-31
African Buffalo	ACA>ATA	0.245676565278257	0.5345831692479	1	1.60197000150362e-20	9.15980365655581e-07	8.14726498131359e-07	4.88041817972547e-23
Water Buffalo	ACA>ATA	0.999999999999997	1	0.5345831692479	4.33358449891738e-13	1.21265352481191e-08	2.80520331405073e-06	5.34596453245024e-11
Cattle	ACA>ATA	1	0.999999999999997	0.245676565278257	2.05453626337933e-14	1.74056907307683e-16	2.37736927137479e-08	1.46341296251644e-20
Cattle	ACC>AAC	1	0.479583346593245	NA	2.53831236357173e-06	NA	3.56267896337184e-10	2.25767239162432e-80
Cattle	ACT>AGT	1	0.0332855811892446	0.720295621758805	6.56386517590871e-06	NA	2.43915549870389e-06	NA
African Buffalo	ACT>ATT	0.68880138218121	0.836101239227671	1	NA	6.83325567471431e-07	NA	2.4073870764137e-24
Water Buffalo	ACT>ATT	0.999999999999997	1	0.836101239227671	NA	7.23426849579628e-06	NA	9.41933678706983e-15
Cattle	ACT>ATT	1	0.999999999999997	0.68880138218121	NA	1.34906957837216e-11	NA	2.45305970768461e-27
Cattle	CAT>CTT	1	0.055267968731054	0.393454559613469	2.29614696934811e-06	NA	1.1283970279302e-22	1.15626270869651e-06
African Buffalo	CCA>CTA	0.246793971259826	0.146704737425378	1	4.81976975372889e-20	NA	NA	1.1224802835464e-08
Cattle	CCA>CTA	1	0.433302524860579	0.246793971259826	2.27177570759421e-14	3.54606336110596e-07	NA	NA
African Buffalo	CCC>CTC	0.00138239203787284	0.00915212492165735	1	9.75817594400095e-06	NA	2.5328855058777e-16	NA
Water Buffalo	CCC>CTC	0.869871461852008	1	0.00915212492165735	4.43913755344232e-10	NA	7.78765914454879e-07	NA
Cattle	CCC>CTC	1	0.869871461852008	0.00138239203787284	1.08766474104199e-13	NA	9.24984618016114e-09	NA
Water Buffalo	CCT>CTT	0.611686625733254	1	0.860938103555499	3.88344201093898e-06	NA	1.04660378911649e-08	NA
African Buffalo	CCT>CTT	0.87117483850567	0.860938103555499	1	7.1334608749608e-06	NA	6.75672492959123e-11	NA
Cattle	CCT>CTT	1	0.611686625733254	0.87117483850567	7.83622310880981e-08	NA	4.70595495199956e-11	NA
Cattle	GAT>GTT	1	0.103892195690961	0.00778234407632577	NA	NA	1.09312284464056e-08	1.93232533521049e-47
Cattle	TCA>TGA	1	0.60332372557456	0.0559543946725239	5.18928536150944e-06	NA	4.4889657910817e-06	NA
African Buffalo	TCA>TTA	0.00267885828896033	0.000153081674499744	1	3.75944176588498e-18	NA	NA	3.21071016301879e-21
Cattle	TCA>TTA	1	0.0496667106787063	0.00267885828896033	5.02562322473407e-11	1.2278357303147e-07	NA	6.49998992070776e-11

Cattle	TCC>TTC	1	0.933197396566466	0.443719832143633	3.05310549425152e-07	6.0616012122907e-06	NA	NA
African Buffalo	TCT>TTT	0.000429230590121764	0.377874829211101	1	2.08881361591201e-14	NA	NA	2.27651713524649e-25
Water Buffalo	TCT>TTT	0.125540706826316	1	0.377874829211101	1.46741583333383e-08	NA	NA	5.42116803631631e-12
Cattle	TCT>TTT	1	0.125540706826316	0.000429230590121764	9.36736784202844e-07	2.84900883425921e-08	NA	1.1009219128869e-12

Supplementary Table 1. Changes that are significantly different between bovids and non-bovids, but not between bovids. In red, the A[C>T]A change that is significantly different between bovids and non-bovids.

COSMIC Signature	Cow	W. Buffalo	A. Buffalo	Horse	Pig	Dog	Human
SBS1	0.50702944	0.5664181	0.52174506	0.63288056	0.30071445	0.56760917	0.4878192
SBS2	0.26213605	0.29140154	0.28665798	0.19009189	0.26651374	0.26733458	0.20194472
SBS3	0.6482302	0.63601792	0.65324069	0.61551521	0.68225387	0.63214399	0.68807835
SBS4	0.27157703	0.28179543	0.30494019	0.27459721	0.27345787	0.29093651	0.29980619
SBS5	0.91808297	0.88194745	0.90252065	0.84428952	0.94517089	0.86733788	0.93357963
SBS6	0.61426229	0.66870561	0.61878376	0.67239575	0.46637959	0.69925565	0.58639013
SBS7a	0.35709482	0.39027455	0.37861339	0.28081387	0.35958705	0.38857052	0.30147392
SBS7b	0.42815073	0.45563629	0.43669371	0.35467265	0.42718744	0.50043644	0.39453688
SBS7c	0.1900938	0.18664369	0.19835717	0.17179721	0.20433164	0.18618891	0.19499287
SBS7d	0.29582805	0.28900496	0.29043054	0.28922419	0.33605395	0.27594018	0.30632856
SBS8	0.31370124	0.31669663	0.34609366	0.31156458	0.32062	0.32270945	0.35289916
SBS9	0.49632851	0.4699978	0.49799061	0.45914066	0.51931185	0.44117682	0.50993318
SBS10a	0.09596288	0.09821925	0.11778019	0.07835767	0.08039223	0.09072339	0.0905816
SBS10b	0.2877199	0.32216301	0.31053751	0.30149346	0.21537697	0.30964441	0.2625295
SBS10c	0.34352885	0.33753818	0.3674503	0.32800881	0.34229548	0.32626372	0.35362833
SBS10d	0.13417213	0.13397467	0.16076419	0.11368653	0.12163839	0.12443495	0.13187996
SBS11	0.44230181	0.46636251	0.45093843	0.35795657	0.46294686	0.50520947	0.41250322
SBS12	0.58332807	0.50749146	0.55163787	0.56454539	0.68590006	0.47687107	0.63198844
SBS13	0.12193598	0.11993996	0.11876133	0.11580649	0.09343383	0.08991645	0.13761991
SBS14	0.20446383	0.2094431	0.22901376	0.21326964	0.19663666	0.20502874	0.2107894
SBS15	0.43796602	0.48033238	0.44324656	0.49743127	0.33361663	0.50533203	0.43041523
SBS16	0.50369786	0.41318093	0.44649059	0.42734006	0.51997242	0.37435121	0.55992431
SBS17a	0.17389061	0.1675998	0.1793583	0.18370795	0.23987425	0.16843421	0.19580485
SBS17b	0.07439728	0.07649586	0.07304845	0.07358331	0.07529083	0.05761478	0.07629954
SBS18	0.29453295	0.30789783	0.33233218	0.291806	0.26519876	0.30504022	0.29642215
SBS19	0.45726136	0.47583378	0.45436555	0.36246363	0.46974186	0.54319792	0.42491747
SBS20	0.3177909	0.32229552	0.32928468	0.31285957	0.32662945	0.33372854	0.32135455

SBS21	0.32196597	0.31237403	0.32521634	0.37366339	0.39647542	0.2970618	0.34988222
SBS22	0.13117791	0.13670633	0.14385022	0.1356757	0.14108775	0.14142651	0.14364397
SBS23	0.44340798	0.46385312	0.43701942	0.3699216	0.45254395	0.54004384	0.43280739
SBS24	0.30031846	0.3153315	0.32265144	0.30741603	0.29127271	0.3314299	0.32863307
SBS25	0.61408589	0.61408366	0.63107931	0.6114942	0.61468274	0.60639336	0.6274597
SBS26	0.59555817	0.53389234	0.57876305	0.60802676	0.70624612	0.50013872	0.64832669
SBS27	0.14543579	0.15192773	0.15442278	0.13424623	0.14262506	0.15544982	0.13872913
SBS28	0.11672281	0.10917743	0.1188607	0.1037392	0.10475251	0.08337767	0.11172631
SBS29	0.26723674	0.28581888	0.30363867	0.29632445	0.23432676	0.2902459	0.29304641
SBS30	0.56925654	0.5999261	0.57604523	0.45322785	0.57687848	0.62671436	0.50475356
SBS31	0.46358278	0.46796601	0.46104477	0.40065122	0.47529235	0.52759124	0.46599842
SBS32	0.57785913	0.60640575	0.59398732	0.49979883	0.57743499	0.62063447	0.52991399
SBS33	0.33590289	0.32213608	0.35431873	0.36583441	0.42424352	0.30528038	0.34453967
SBS34	0.09100515	0.09614903	0.10040001	0.08290867	0.08217134	0.09722505	0.09550313
SBS35	0.29098027	0.30442311	0.31056667	0.28106581	0.29360321	0.33975309	0.3200253
SBS36	0.20019804	0.20552569	0.23436345	0.18595579	0.18212006	0.1990691	0.20129578
SBS37	0.55767749	0.49206679	0.52226317	0.53089209	0.61380029	0.45810816	0.59813534
SBS38	0.11054605	0.11472928	0.12889225	0.11920201	0.10747781	0.11240456	0.11727754
SBS39	0.46329967	0.45906185	0.45762701	0.47385263	0.4457286	0.42823585	0.50209969
SBS40	0.69393904	0.68316281	0.70719007	0.61831919	0.7229701	0.66512185	0.7017572
SBS41	0.41259025	0.39170672	0.41039156	0.37938575	0.41130167	0.36810259	0.42036911
SBS42	0.44425813	0.45759282	0.45073059	0.39308268	0.47046438	0.51011623	0.45988276
SBS43	0.14607451	0.14337718	0.15413544	0.15188791	0.16766768	0.13592627	0.15117276
SBS44	0.58590454	0.58871428	0.58910721	0.55814867	0.60558364	0.60496952	0.57824463
SBS45	0.11155854	0.11755232	0.13518556	0.12336965	0.10479129	0.1158085	0.12229538
SBS46	0.56480154	0.53633476	0.55875211	0.55176553	0.66415856	0.5207392	0.5802435
SBS47	0.21445592	0.22392815	0.23638007	0.18896509	0.21642622	0.21957519	0.20507385
SBS48	0.02316254	0.02884337	0.02642033	0.03294519	0.01857374	0.02579404	0.02408477
SBS49	0.04411558	0.0518224	0.05166456	0.06393733	0.04007556	0.04903691	0.04995513
SBS50	0.30425881	0.32076606	0.33438138	0.28497293	0.30939334	0.32487359	0.30869721

SBS51	0.29890604	0.30969747	0.32033528	0.2870376	0.30016246	0.30784769	0.29906871
SBS52	0.09548648	0.09795425	0.11446415	0.08768812	0.08355876	0.09148637	0.09175995
SBS53	0.16932744	0.1721882	0.17776058	0.17907631	0.16454476	0.17189972	0.17686753
SBS54	0.51391045	0.4753473	0.52844191	0.54462164	0.61338191	0.4506867	0.54740201
SBS55	0.1581108	0.16411318	0.17368874	0.15769226	0.15630159	0.15126008	0.16316758
SBS56	0.1291213	0.12692222	0.15324573	0.10852144	0.11292903	0.11606236	0.1267718
SBS57	0.43454363	0.40933898	0.42623332	0.3821074	0.44574465	0.39975538	0.43468884
SBS58	0.50479545	0.49714149	0.51035323	0.42902089	0.51735681	0.48614	0.48298208
SBS59	0.08424515	0.08521085	0.09759544	0.09210121	0.08799901	0.08729326	0.09573626
SBS60	0.06127123	0.06408813	0.07079878	0.06299791	0.06142825	0.05855382	0.06119462
SBS84	0.43229059	0.44730445	0.42837644	0.3717118	0.4512519	0.49278019	0.42345807
SBS85	0.35877891	0.32820219	0.34808745	0.31869502	0.38581762	0.30943782	0.37773891
SBS86	0.29136099	0.29879564	0.28741299	0.31110634	0.24243993	0.2658487	0.31505034
SBS87	0.53292952	0.58062599	0.54067015	0.62898983	0.3615481	0.59042219	0.52027401
SBS88	0.41838217	0.34361596	0.37134829	0.34509259	0.41603842	0.3079604	0.45248127
SBS89	0.57942115	0.5772302	0.59639861	0.53382581	0.59275484	0.56293258	0.58668304
SBS90	0.0788636	0.08156822	0.08862219	0.06819053	0.07747707	0.07733549	0.07314013
SBS91	0.1033347	0.1101165	0.11988125	0.08892411	0.09382132	0.10489773	0.09593333
SBS92	0.77683088	0.73020623	0.75191475	0.70242861	0.81044402	0.72765413	0.80875062
SBS93	0.49152912	0.47090141	0.48984136	0.4773825	0.49057635	0.44467357	0.5148775
SBS94	0.52493379	0.53252741	0.54148095	0.51947563	0.50027599	0.5431748	0.55009699
SBS95	0.3482759	0.36151506	0.38509482	0.34457447	0.35436781	0.37333369	0.36580181

Supplementary Table 2. Cosine similarities between the signatures for the different species with the COSMIC database.

Species	Breed	Genome	Length	Number of Sequences
Sus scrofa		GCF_000003025.6	2501912388	613
Capra sibirica		GCA_003182615.2	2733031394	85609
Capra hircus	San Clemente	GCF_001704415.2	2922813246	29907
Capra hircus	Pashmina goat	GCA_009823495.1	2941702774	48370
Capra hircus	Yunnan black	GCA_000317765.2	2809418462	102895
Capra aegagrus		GCA_000978405.1	2583320943	6616
Capra aegagrus		GCA_000765075.1	2828873048	89498
Capra hircus	Black Bengal goat	GCA_004361675.1	3041623660	3972
Capra ibex		GCA_006410555.1	2701356656	55914
Bos grunniens		GCA_005887515.3	2832776395	414
Bos taurus	Ankole	GCA_905123885.1	2921040163	7581
Bos taurus	Brahman	GCA_003369695.2	2680969395	1251
Bos taurus	NDama	GCA_905123515.1	2766829411	1210
Bos taurus	Hereford	GCF_002263795.1	2759153975	2212
Bos taurus	Angus	GCA_003369685.2	2580764822	1435
Bubalus bubalis		GCF_003121395.1	2655780776	509
Syncerus caffer		GCA_902825105.1	2652967596	3351

Supplementary Table 3. List of genomes and accession numbers used to generate the alignments with progressive cactus.
