# Supplementary material for: The evolution and convergence of mutation spectra across mammals
Source: Commun Biol. 2025 May 17;8:763. doi: 10.1038/s42003-025-08181-x (PMC12084637; doi:10.1038/s42003-025-08181-x)
Supplement: Supplementary file 3 — Description of Additional Supplementary Files [file 42003_2025_8181_MOESM3_ESM.pdf]

Description of Additional Supplementary Files

File Name: Supplementary Data 1
Description: The full list of pairwise comparisons of mutations between species with their P-values.

File Name: Supplementary Data 2
Description: The data underlying the figures.
